# Chronic Eosinophilic Leukemia Not Otherwise Specified: A Case Series

**DOI:** 10.14740/jh2114

**Published:** 2026-06-20

**Authors:** Neilmegh Lakshman Varada, Katherine Dougherty Bordes, Jordan P. Redemann, Ala Ebaid

**Affiliations:** a Department of Internal Medicine, University of New Mexico Comprehensive Cancer Center, Albuquerque, NM, USA; b Tulane University School of Medicine, New Orleans, LA, USA; c Department of Pathology, University of New Mexico Comprehensive Cancer Center, Albuquerque, NM, USA

**Keywords:** Chronic eosinophilic leukemia, Hypereosinophilia, Myeloproliferative neoplasm

## Abstract

Chronic eosinophilic leukemia (CEL) is a rare myeloproliferative neoplasm characterized by sustained elevation of eosinophil counts greater than > 1.5 × 10^9^/L in blood or bone marrow. Approximately 25–30% of patients with persistent hypereosinophilia have somatic mutations associated with myeloid neoplasms, and next generation sequencing has led to the use of newer treatments for CEL, including tyrosine kinase inhibitors (TKIs) such as imatinib. Before the advent of imatinib, the disease had a poor prognosis with a 5-year mortality close to 50%. However, in patients with CEL without the characteristic mutations, known as chronic eosinophilic leukemia, not otherwise specified (CEL-NOS), treatment options and guidance are limited. We present a case series of two CEL-NOS patients treated at our academic health sciences center.

## Introduction

Chronic eosinophilic leukemia (CEL) is a rare myeloproliferative neoplasm characterized by sustained elevation of eosinophil counts greater than > 1.5 × 10^9^/L in blood or bone marrow. The International Consensus Classification (ICC) Diagnostic Criteria for CEL include sustained hypereosinophilia, blasts constituting < 20% of cells in peripheral blood and bone marrow, a lack of tyrosine kinase fusion mutations, and increased cellularity often with dysplastic megakaryocytes and an eosinophilic infiltrate in bone marrow [1–3]. The fifth edition of the World Health Organization Classification of Hematolymphoid Tumors defines CEL as persistent eosinophilia with an absolute eosinophil count of greater than 1.5 × 10^9^, evidence of clonality with cytogenetic abnormalities, molecular mutations, or increased blasts [4]. Approximately 25–30% of patients with persistent hypereosinophilia have somatic mutations associated with myeloid neoplasms. These include tyrosine kinase gene fusions such as *PDGFRA*, *PDGFRB*, *FGFR1*, *JAK2, ABL1*, and *FLT3*, somatic mutations such as *TET2*, *ASXL1*, *EZH2*, and *SETBP1*, and activating *STAT5b N642H* mutations. Next-generation sequencing has identified positive mutations in some patients without evidence of myeloid neoplasm, such as idiopathic hypereosinophilic syndrome (iHES) or hypereosinophilia of unknown significance (HEus). In contrast, some patients have evidence of myeloid neoplasm but no detectable mutations. Although the strength of association between CEL and genetic mutations has yet to be determined, molecular genetics plays an essential role in identifying potential therapies, such as tyrosine kinase inhibitors (TKIs), and in ruling out other hematologic neoplasms or dyscrasias [1, 5].

Fusion genes such as *FIP1L1::PDGFRA*, *ETV6::ABL1*, and *BCR::ABL1* help differentiate myeloid/lymphoid neoplasms with eosinophilia from CEL. In terms of non-neoplastic hypereosinophilia, iHES and HEus are closely related differential diagnoses for CEL due to the clinicopathologic overlap. The key differentiating factor for iHES is the presence of a cytologic or molecular genetic alteration, whereas for HEus, it is a lack of organ damage and dysfunction. Other causes of eosinophilia, including reactive processes and systemic mastocytosis, must also be excluded [1, 2, 5].

With regard to patient demographics and clinical presentation, patients with CEL are typically older and may be asymptomatic. If associated symptoms are present, they are typically nonspecific, such as fever, chills, night sweats, weight loss, and skin and mucosal lesions. Over time, hypereosinophilia can lead to organ damage and degradation due to inflammatory processes triggered by the release of cytokines related to T-cell activity. Organs that may be involved include the heart (endomyocardial fibrosis, restrictive cardiomyopathy, and valvular dysfunction), nervous system (peripheral neuropathy and central nervous system dysfunction), gastrointestinal tract (hepatosplenomegaly, diarrhea), and lung (pulmonary infiltrates) [2].

Treatment is often successful with TKIs, such as imatinib, although certain mutations can bypass the site of drug activity. Here, we present two cases of CEL who were treated in an academic health sciences center.

## Case Reports

### Case 1

#### Patient history

Our first patient is a 77-year-old male who presented to the hematology oncology clinic for evaluation due to eosinophilia in 2020, with a past medical history of congestive heart failure, diverticulosis, hypertension, and hyperlipidemia. He underwent an initial examination through his primary care provider due to findings of eosinophilia, with an absolute eosinophil count of 9.4 and a total white blood cell (WBC) count of 32.3 with elevated eosinophils, which was confirmed by a review of his known labs dating back to 2017. Notably, he was asymptomatic upon presentation, except for allergy-like symptoms, including a runny nose and congestion. He denied any recurrent infection, abnormal bleeding or bruising, bone pain, unexplained fever, or night sweats. Differential diagnosis of hyper-eosinophilic syndrome was considered, and further workup was prompted. Testing for the *FIP1L1::PDGFRA* fusion was negative, but an abdominal ultrasound demonstrated borderline splenomegaly, and a complete blood cell (CBC) with differential showed an increase in his mast cells. Bone marrow biopsy was planned, but was lost to follow-up till 2024. He presented again due to worsening symptoms of fatigue and night sweats. As a result, it was recommended he undergo a bone marrow biopsy.

#### Diagnostic workup

**Figure 1:**
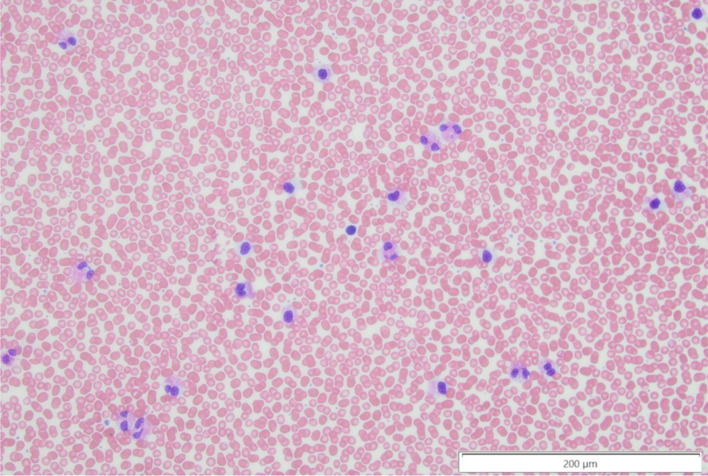
Peripheral blood smear with dysplastic eosinophils, noting the abnormal nuclear contours in a subset of the eosinophils and the atypical uneven granulation at × 20 magnification.

**Figure 2:**
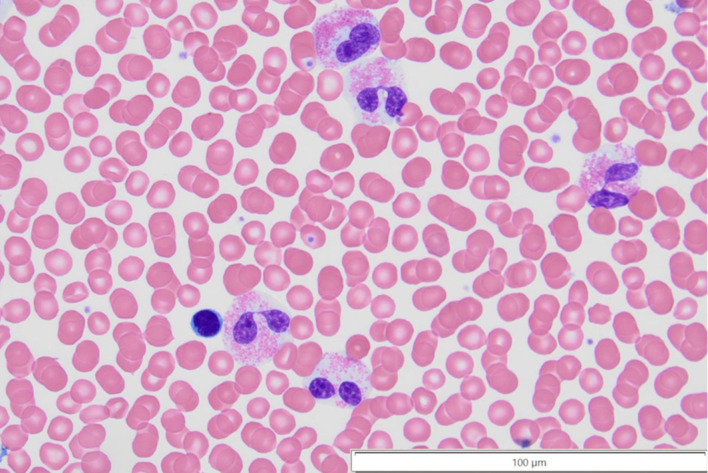
Peripheral blood with dysplastic eosinophils, noting the completely separated nuclear lobes, and the cytoplasm with less than 50% of typical granulation in the bottom most eosinophil at × 60 magnification.

**Figure 3:**
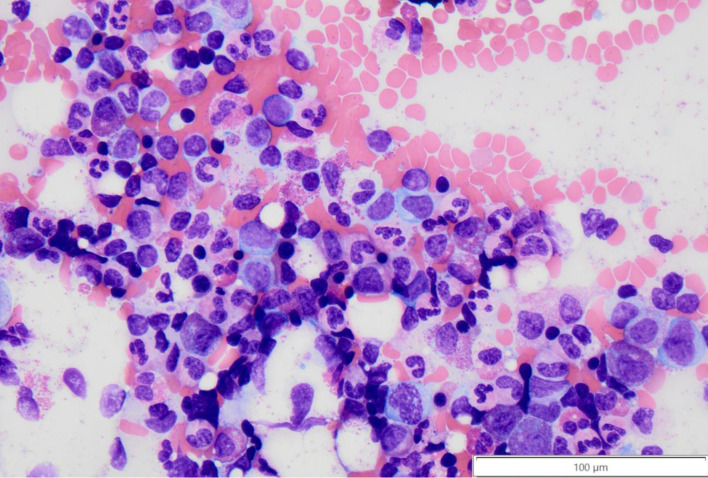
Bone marrow aspirate smear with focally increased eosinophil precursors with uneven granulation at × 40 magnification.

**Figure 4:**
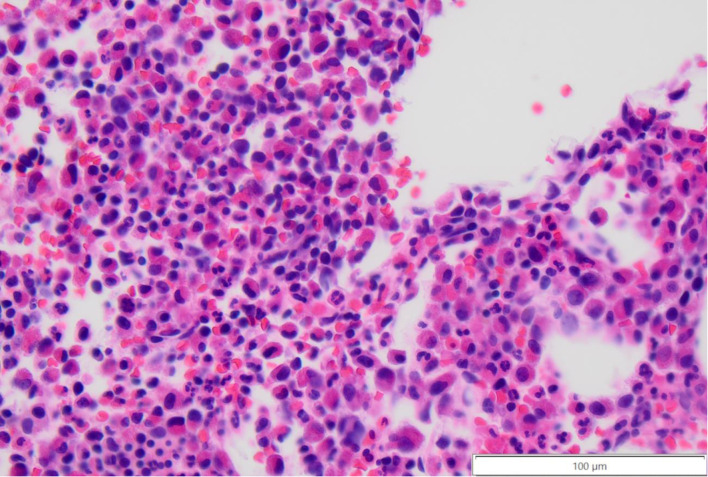
Core bone marrow biopsy with increased eosinophil precursors (mononuclear to bilobed cells with deep eosinophilic cytoplasm) at × 40 magnification.

Further workup included a bone marrow biopsy performed via interventional radiology and a peripheral blood smear. Lab workup at the time demonstrated his WBC elevated at 32.3 × 10^3^/µL with an eosinophil count of 9.4 and a tryptase that was elevated at 15.3 ng/mL (Table 1). The smear, as seen in Figures 1 and 2, demonstrated eosinophilia with dysplastic eosinophils and lymphocytosis. The bone marrow biopsy results showed hypercellular marrow, myeloid predominant trilineage hematopoiesis, slightly increased blasts of less than 5%, increased eosinophils, and increased scattered mast cells as seen in Figures 3 and 4. Further testing demonstrated negative for *BCR::ABL1* rearrangement. Flow cytometry did not show an increase in mast cells, CD34 cells, or monotype B or T cells, with a negative C-KIT digital PCR result. However, his myeloid gene panel showed mutations in *JAK2* c.1748_1757delinsA, p.Leu583_Ala586delinsGln with a variant allele frequency (VAF) of 15.5% and a JAK3 mutation with a VAF of 46.4%, and a chromosome analysis demonstrated a deletion of the Y chromosome. Fluorescence *in situ* hybridization (FISH) for eosinophilia was negative for the PDGFRB, FGFR1, and JAK2. Karyotype analysis demonstrated 45,X,-Y (5)/46,XY (15). Kit mutational analysis for mastocytosis was also negative. Overall, given the sustained eosinophilia, bone marrow with elevated eosinophils, and JAK2 exon 13 mutations without any tyrosine kinase gene fusion, the patient was diagnosed with CEL. Treatment with a TKI, such as imatinib, was considered.

**Table 1:** Lab Values of Case 1

	2020 (original consultation)	2024 (second consultation)	2024 (starting on TKI)	2024 (1 month after TKI)	2025 (3 months after TKI)
White blood cell count	18.1 × 10^3^/µL	32.3 × 10^3^/µL	19.5 × 10^3^/µL	24.8 × 10^3^/µL	26.0 × 10^3^/µL
Absolute eosinophil count	9.1 × 10^3^/µL	9.4 × 10^3^/µL	11.6 × 10^3^/µL	13.8 × 10^3^/µL	14.6 × 10^3^/µL
Hemoglobin	15.3 g/dL	14.9 g/dL	13.2 g/dL	13.5 g/dL	12.7 g/dL
Platelet count	160 × 10^3^/µL	171 × 10^3^/µL	138 × 10^3^/µL	135 × 10^3^/µL	146 × 10^3^/µL

TKI: tyrosine kinase inhibitor.

#### Treatment

The patient’s case was discussed at tumor board. During this time, the patient developed progressive eosinophilia and constitutional symptoms, mainly night sweats and fatigue. Due to his findings and similarities to mastocytosis, namely his symptoms, the patient was initiated on imatinib 100 mg qd. Imatinib is effective for classic CEL subtypes with PDGFRA and PDGFRB rearrangement, but is generally ineffective for CEL driven by JAK family kinases. This drug was chosen due to clinical similarities to mastocytosis and published evidence of its efficacy in some PDGFRA and PDGFRB-negative CEL cases [6].

#### Outcome

This resulted in a significant improvement in his symptoms. However, hematological parameters, including his WBC, hemoglobin, and platelet count remained stable as seen in Table 1. His only side effect was a transient rash on the anterior surface of his forearms, which resolved spontaneously. Rash is a common side effect from imatinib. It is believed to be secondary to the inhibition of the c-Kit receptor tyrosine kinase, which can also be found in skin cells. Mild cases are often seen and can resolve spontaneously [7]. The WBC and absolute eosinophil count, however, remained elevated, with his last WBC being 26 and eosinophil count at 18.2.

### Case 2

#### Patient history

Our second patient is a 46-year-old male with a past medical history of eosinophilic granulomatosis with polyangiitis and Ebstein anomaly, who presented for evaluation after being diagnosed with chronic eosinophilic leukemia in 2013 at an outside hospital. Bone marrow biopsy and aspirate obtained at the time showed CEL associated with *FIP1L1::PDGFRA* fusion and MF-1 fibrosis. At the time, he started imatinib 100 mg daily.

#### Diagnostic workup

A bone marrow biopsy was repeated 1 year later, and the blast count had increased to 8%. Curiously, during this time, he presented with headaches, and imaging demonstrated concerns for vasculitis. He was diagnosed with eosinophilic granulomatosis with polyangiitis, which was confirmed with a brain biopsy. He was then treated with four weekly infusions of rituximab, after which it was slowed to rituximab infusion every 6 months, for a total of 2 years, combined with prednisone 10 mg PO daily. The patient’s symptoms had improved significantly; he remained on the imatinib, and his prednisone was tapered off. Notably in 2023, the patient also was admitted for evaluation of left-sided deficits, which was found to be a cerebrovascular accident (CVA).

However, in 2025, the patient developed lower extremity swelling and went to the emergency room for further evaluation. He was found to be in atrial fibrillation and acute congestive heart failure. He was evaluated by the cardiology team, who recommended discontinuation of his imatinib.

#### Treatment

On return to the hematology clinic, given his lack of symptoms and new contraindication to imatinib, a repeat bone marrow biopsy was conducted. The biopsy curiously noted mildly hypocellular bone marrow with no morphologic immunophenotype, or FISH of the previously diagnosed myeloid neoplasm with PDGFRA rearrangement.

#### Outcome

He has remained off his imatinib and is now being actively monitored without any symptoms. His counts have also remained stable as seen in Table 2.

**Table 2:** Lab Values of Case 2

	2017 (original consultation)	2023 (CVA)	2025 (atrial fibrillation diagnosis and stopped TKI)	2025 (1 month after cessation of TKI)	2025 (5 months after TKI)
White blood cell count	11.7 × 10^3^/µL	8.9 × 10^3^/µL	7.5 × 10^3^/µL	7.7 × 10^3^/µL	7.2 × 10^3^/µL
Absolute eosinophil count	0.1 × 10^3^/µL	0.1 × 10^3^/µL	0.2 × 10^3^/µL	0.2 × 10^3^/µL	0.2 × 10^3^/µL
Hemoglobin	17.1	16.4	17.3	18.5	16.5
Platelet count	173	172	167	164	160

CVA: cerebrovascular accident; TKI: tyrosine kinase inhibitor.

## Discussion

CEL is a rare condition. Before the advent of imatinib, the disease had a poor prognosis with a 5-year mortality close to 50% [8]. Imatinib acts on the FIP1L1/PDGFRA fusion gene in a more sensitive way than its actions on BCR/ABL [9]. Thus, imatinib is a significant breakthrough medication in the treatment of CEL, as seen in the second case.

CEL is defined according to the 2022 WHO classification criteria. The diagnostic requirements are as follows: persistent eosinophilia, with an eosinophil count greater than 1.5 × 10^9^/L seen in peripheral blood on two examinations 4 weeks apart; exclusion of other WHO-defined hematologic neoplasms such as myelodysplastic syndrome (MDS), myeloproliferative neoplasm (MPN), systemic mastocytosis, and acute myeloid leukemia (AML); absence of MLN-TK rearrangements such as *PDGFRA*, *PDGFRB*, *FGFR1*, *ETV6::ABL1*, *JAK2*, and *FLT3*; presence of clonal cytogenetic or molecular genetic alterations; and detection of an aberrant morphology in the bone marrow [4].

Tyrosine kinases are a class of enzymes that induce signal transduction through phosphorylation of amino acids on substrate proteins. This signal transduction leads to cellular changes, including growth, differentiation, death, and constitutive activation. In the setting of malignancy and myeloproliferative diseases, blocking these signals from tyrosine can induce downstream apoptosis without further differentiation [10]. TKIs, such as imatinib, target platelet-derived growth factor receptor (PDGFR), c-kit, and BCR-ABL fusion protein. Though revolutionary in its inhibition of BCR-ABL, the wild type C-ABL can also be inhibited by imatinib. This inhibition is the theorized reason behind the cardiotoxicity seen in TKIs. Distler and Distler noted that congestive heart failure may be induced by imatinib, but its rate is low, but also noted that the incidence of cardiotoxic side effects may occur in those with pre-existing cardiac disease [11]. Case 1 exhibits the tolerance of imatinib for patients with heart failure. Imatinib was also trialed in the patient in case 2, but discontinued due to the development of atrial fibrillation and heart failure.

A study by Cools et al examined mutations in patients with hypereosinophilic syndrome. They found that some patients had a fusion protein, FIP1L1-PDGFRα, which reclassified their diagnosis as CEL, based on WHO guidelines at the time of the study. This FIP1L1-PDGFRα mutation transformed a murine hematopoietic cell line Ba/F3 to interleukin–3–independent growth and was constitutively tyrosine-phosphorylated in these cells. They tested the effect of imatinib on Ba/F3 cells expressing FIP1L1-PDGFRα and determined that they were more efficiently inhibited by much lower concentrations of imatinib than Ba/F3 cells expressing the BCR-ABL, 3.2 and 582 nM, respectively. These findings show that imatinib is highly specific in targeting the PDGFR kinase domain [9].

However, in CEL without the *FIP1L1::PDGFRA* fusion gene, patients fare far worse. This form of the disease is termed CEL-NOS, defined as CEL with the absence of the Philadelphia chromosome or a rearrangement involving PDGFRA, PDGFRB, or FGFR1 rearrangements and PCM1-JAK2, ETV6-JAK2, or BCR-JAK2 fusion genes, and the exclusion of other acute or chronic primary marrow neoplasms associated with eosinophilia [12]. In some case series, the prognosis in these patients was poor, with a median survival of 22 months, a portion of whom converted to acute leukemia [12].

However, the 2022 WHO guidelines included a comment on the rare instance where imatinib-based therapies for CEL-NOS can result in a hematologic response. In their remarks, a higher dose, greater than 400 mg, was recommended, unlike our treatment of 100 mg as seen in case 1. Significantly, they noted that this may be a partial, short-lived response, and could represent either drug-related myelosuppression or diagnostically occult PDGFRA or PDGFRB mutations or another target [4]. Our patient continues to demonstrate notable suppression of all symptoms and no signs of progression at the current 6-month mark.

Iurlo et al demonstrated successful treatment in CEL-NOS, with a M541L c-KIT mutation, using imatinib treatment, which resulted in a rapid hematologic remission [13].

Case 1 also exemplifies the heterogeneity in the response sometimes seen with imatinib. As Loscocco et al noted though imatinib works with PDGFRA-positive disease, it has some effect in PDGFRA/B-negative disease in some patients, and in their prospective trial, an overall and complete hematological response rate was seen in 46.9% and 18.8% of patients treated with imatinib [14]. It was postulated that another genetic mutation may be present in this subset of patients, but also noted that the response was short-lived in most patients.

The use of TKIs in CML was a revolutionary change in therapy. However, their long-term use has been associated with side effects in this population. Likewise, their further use in diseases like CEL has led to discussions on the length of treatment. We demonstrate two approaches: long-term maintenance treatment and discontinuation of therapy once remission is achieved. The length of treatment and monitoring after the disease is not well established, as Klion et al mentioned. Molecular monitoring after remission, and potential “cure,” can be used, as seen in case 2 above [8].

CEL remains a rare disease; however, with further next-generation sequencing studies being used, the diagnosis of more cases of CEL is possible, both traditional and NOS [15]. Evaluating and identifying new mutations will also be important. These mutations may result in helping predict new indications for therapies such as imatinib, and assisting in development of new medications all together. As such, consideration may be given to the use of tyrosine kinase inhibitors, even in patients with CEL-NOS.

## Data Availability

The authors declare that data supporting the findings of this study are available within the article.

## References

[1] Arber DA, Orazi A, Hasserjian RP, Borowitz MJ, Calvo KR, Kvasnicka HM, Wang SA (2022). International consensus classification of myeloid neoplasms and acute leukemias: integrating morphologic, clinical, and genomic data. Blood.

[2] Wahed A, Dasgupta A, Wahed A, Dasgupta A (2015). Chapter 6 - myeloid neoplasms. Hematology and coagulation.

[3] Lierman E, Michaux L, Beullens E, Pierre P, Marynen P, Cools J, Vandenberghe P (2009). FIP1L1-PDGFRalpha D842V, a novel panresistant mutant, emerging after treatment of FIP1L1-PDGFRalpha T674I eosinophilic leukemia with single agent sorafenib. Leukemia.

[4] Khoury JD, Solary E, Abla O, Akkari Y, Alaggio R, Apperley JF, Bejar R (2022). The 5th edition of the World Health Organization Classification of Haematolymphoid Tumours: Myeloid and Histiocytic/Dendritic Neoplasms. Leukemia.

[5] Tzankov A, Reichard KK, Hasserjian RP, Arber DA, Orazi A, Wang SA (2023). Updates on eosinophilic disorders. Virchows Arch.

[6] Morsia E, Reichard K, Pardanani A, Tefferi A, Gangat N (2020). WHO defined chronic eosinophilic leukemia, not otherwise specified (CEL, NOS): a contemporary series from the Mayo Clinic. Am J Hematol.

[7] Kataria P, Patel A, Kendre P, Tahiliani N, Mule T, Bohra M (2022). Erythroderma: an unusual manifestation of imatinib - A rare case report. J Cancer Res Ther.

[8] Klion AD, Robyn J, Maric I, Fu W, Schmid L, Lemery S, Noel P (2007). Relapse following discontinuation of imatinib mesylate therapy for FIP1L1/PDGFRA-positive chronic eosinophilic leukemia: implications for optimal dosing. Blood.

[9] Cools J, DeAngelo DJ, Gotlib J, Stover EH, Legare RD, Cortes J, Kutok J (2003). A tyrosine kinase created by fusion of the PDGFRA and FIP1L1 genes as a therapeutic target of imatinib in idiopathic hypereosinophilic syndrome. N Engl J Med.

[10] Gambacorti-Passerini C, le Coutre P, Mologni L, Fanelli M, Bertazzoli C, Marchesi E, Di Nicola M (1997). Inhibition of the ABL kinase activity blocks the proliferation of BCR/ABL+ leukemic cells and induces apoptosis. Blood Cells Mol Dis.

[11] Distler JH, Distler O (2007). Cardiotoxicity of imatinib mesylate: an extremely rare phenomenon or a major side effect?. Ann Rheum Dis.

[12] Helbig G, Soja A, Bartkowska-Chrobok A, Kyrcz-Krzemien S (2012). Chronic eosinophilic leukemia-not otherwise specified has a poor prognosis with unresponsiveness to conventional treatment and high risk of acute transformation. Am J Hematol.

[13] Iurlo A, Fracchiolla NS, Ferla V, Cassin R, Gottardi E, Beghini A, Gianelli U (2014). Successful treatment with imatinib in a patient with chronic eosinophilic leukemia not otherwise specified. J Clin Oncol.

[14] Loscocco GG, Helbig G (2024). Imatinib is effective in some PDGFRA/B-negative hypereosinophilic syndromes: A step closer to unveiling underlying mechanisms. Br J Haematol.

[15] Kuang FL (2020). Approach to Patients with Eosinophilia. Med Clin North Am.

